# The Impact of Home and Community-Based Services on the Health Status of Older Adults in China: A Longitudinal Analysis

**DOI:** 10.1093/geroni/igaf122.2839

**Published:** 2025-12-31

**Authors:** Shibin Yan

**Affiliations:** University of Pennsylvania, Camden, New Jersey, United States

## Abstract

With China’s rapidly aging population, ensuring the well-being of older adults has become a pressing concern. Home and community-based services (HCBS) play a crucial role in supporting older individuals, particularly those with chronic conditions, by fostering social engagement and promoting healthier living environments. While research has examined self-rated health among older adults, limited attention has been given to how these services influences health outcomes. Moreover, the impact of HCBS on vulnerable subgroups, such as childless and disabled older adults, remains underexplored. This study addresses this gap by analyzing Chinese Longitudinal Healthy Longevity Study (CLHLS) panel data from 2008 to 2018. Using person fixed-effect regression models, the study assesses how HCBS affects health status, measured through self-rated health, interviewer-rated health, and comparative health assessments. Unlike prior studies relying solely on self-rated health, this approach provides a more comprehensive understanding of older adults’ well-being. Findings indicate a significant positive association between HCBS and improved health outcomes. Among various service types, cultural and social services exhibit the strongest effect. The results further reveal disparities based on residential location, disability status, and financial stability—with urban residents benefiting more than rural counterparts. However, no evidence suggests that childlessness moderates this relationship. This research enhances aging studies by utilizing longitudinal models and multidimensional health assessments. Findings highlight the importance of family financial support and HCBS in promoting healthy aging, offering valuable insights for policymakers.

